# Phage Diversity for Research and Application

**DOI:** 10.3390/antibiotics9110734

**Published:** 2020-10-26

**Authors:** Christine Rohde, Johannes Wittmann

**Affiliations:** Leibniz Institute DSMZ, German Collection of Microorganisms and Cell Cultures GmbH, 38124 Braunschweig, Germany; Christine.Rohde@dsmz.de

Bacteriophages (in short, phages) are viruses that specifically recognize and infect bacteria; they are the most abundant forms of life in the biosphere outnumbering bacteria by an estimated factor of one order of magnitude [1]. Logically, lytic phages have an enormous potential in human and veterinary medicine as “intelligent antibiotics”. In contrast to static antibacterial drugs, phages self-control their numbers at the locus of bacterial infection as they replicate on the unwanted pathogen and disintegrate into their subunits when the bacterial host is eliminated. In One-Health approaches addressing the interconnection of human and veterinary medicine including animal farming and environmental health, phages represent the most powerful avenue as alternatives to antibiotics and for remediating environmental drug loads, the latter being a huge sustainable problem [2]. In the antibiotic crisis where bacterial multi-drug resistance and even pan-drug resistance dramatically increase, phages have resurfaced as a promising natural option to fight this global dilemma. However, this rediscovery implies that phages have come into focus of interest for general research again. Despite phages having been used for more than a century, state-of-the-art phage research is required not only in the case of systematic clinical trials but in all fields of phage application. Research will benefit from knowing more on phage diversity and taxonomy, their mode of host interaction, their structure–function correlations and their genotype–phenotype interrelations. Apart from virulent lytic phages, temperate phages and lysogeny and research into phages’ lysogeny decision is highly interesting [3].

Projects analyzing the metagenomes of different habitats already revealed that phage diversity is huge; there is always something new to discover with regard to habitat diversity and to bacterial species still underrepresented in terms of phage research. Lytic and especially temperate phages are leading drivers of evolution and powerfully contribute to biodiversity in the microbe world, including human and animal microbiomes, plants and the rhizosphere or marine and freshwater habitats [4].

In addition to their impact on ecology and evolution, phages have also played an outstandingly important role in the development of molecular biology; they had been the focus of the work by S. E. Luria, M. Delbrueck and A. D. Hershey on bacterial mutation frequency resulting in the Nobel Prize Award for Physiology or Medicine in 1969. The Nobel Prize for Chemistry in 2018 was awarded on substantial findings by F. H. Arnold, G. P. Smith and G. P. Winter on directed evolution of enzymes and binding proteins using phage display. Research is going on to find new ways to efficiently use phage diversity for different applications and phages as tools. Phages are a success story of nature and have served as appreciated model systems, including for human disease such as, e.g., Alzheimer’s disease, AIDS and now in 2020, the SARS-CoV-2 pandemic. However, in the midst of this pandemic, Eisenmann [5] has published a retrospect view on the “Arrowsmith” story written by S. Lewis in 1925, shortly after the 1918 influenza pandemic. Phages for the first time had been the center of attention in a breathtaking book and, later, in a Nobel Prize Award for Literature. Like a virus pandemic, the antimicrobial resistance (AMR) crisis spreads across all borders.

This Special Issue of Antibiotics has drawn together contributions in the fields of phage diversity for research and application and initiates new think tanks towards promoting infrastructures and funding bodies. General phage diversity and different aspects of phage biology and application have been addressed.

Focusing on the rehabilitation of phage therapy, Gabard [6] compares regulatory requirements for production and quality regulations in different therapies that apply living treatments from living organisms (biologicals), especially cell therapy, fecal matter transfer (FMT), bacterial therapy and phage therapy. The author especially provides robust arguments to address prophage content issues in phage API preparations (active pharmaceutical ingredients) from a regulatory perspective and rebalances potential risks associated with the presence of prophages in API preparations in comparison to such phages contained in various therapeutic anti-infective treatments or in food or probiotics. The author explains the French Regulatory Agency (ANSM, Agence nationale de sécurité du médicament et des produits de santé) ranking of prophage risks in five classes which at least offers a basis for suitable risk criteria in the single case. Importantly, the author discusses the ratio between the benefit of phage therapy compared to the risk associated with batch content prophage contamination. 

One important point that has to be kept in mind when discussing phage therapy is that phages should not generally replace antibiotic drugs in therapy but complement their actions and finally strengthen the antibacterial effect on the whole, as expressed by many experts. Often, it may be satisfactory to reduce the load of a bacterial pathogen with both antibacterial strategies together. Of course, it is highly important to work out in the single therapy case how to combine phages and antibiotics to obtain optimal synergistic results. In the AMR crisis, well-balanced combined strategies to combat bacterial pathogens get extremely important and phage collections should include broad phage diversity against the most worrying pathogens but also against others. Weber et al. [7] address this point with a focus on *Serratia marcescens* and on intrinsic antibiotic resistances such as the widespread AmpC β-lactamases. The authors show that *S. marcescens* could be completely eradicated and the emergence of phage-resistant variants suppressed when a newly isolated podovirus and the antibiotic ampicillin/sulbactam were applied together in vitro.

The excessive misuse of antibiotics, in particular in animal farming, led to the wide distribution of multidrug resistant strains, in particular among zoonotic bacteria of the ESKAPE group, and sustainable environmental loads of antibacterial drug resistance genes. This AMR crisis in the context of the so-called One-Health approach in the triad of human, animal and environmental health is brought to attention by Garvey’s overview article [8] where the specificity and potency of phages, as well as their biocompatibility as natural antibacterials. are pictured as highly welcome features of phages. Nevertheless, some weak points in broader phage application that are noted by the author must be overcome, e.g., phage large-scale production and associated questions on purity of phage preparations, formulation, stability and development of bacterial phage resistance and in-depth phage characterization with regard to their genomes and phenotypic properties. The article provides example lists of well-described phages and their characteristics and of bacterial pathogens of the WHO list and calls for interdisciplinary research towards phage application in the broad field of One-Health.

One of those fields that act as a significant target of mass antibiotic drug application is poultry breeding. In that, it is not salmonellosis but campylobacteriosis that is the leading cause of human bacterial gastroenteritis after consumption of poultry and with *Campylobacter jejuni* and *Campylobacter coli* as causative bacteria. Lynch et al. analyzed a large number of broiler samples to detect antimicrobial resistance determinants in detail and found both in *Campylobacter*, horizontal gene transfer by mobile genetic elements and chromosomally located resistant lineages [9]. A very high prevalence of antimicrobial resistance was found in the *Campylobacter* samples so that these findings were called a major societal challenge by the authors, especially the prevalence of (fluoro)quinolones and tetracycline resistances, with DNA gyrase in Gram-negatives being the main target of fluoroquinolones. The analyses in this whole-genome project shed light on different antibiotic resistance determinants by investigating the mutation loci involved and hence on the situation of the highly critical AMR prevalence in *Campylobacter* and the broiler industry. 

One detailed example for the potential use of phages in a One-Health context is presented in the publication by Le et al. [10]. The authors have successfully applied an anti-Vibrio three phage cocktail as a natural bio-control in oyster hatcheries. For many years, the idea to protect aquaculture hatcheries by using phages against unwanted bacterial pathogens has been raised. In the center of interest are, among others also, different species of *Vibrio*. Usage of antibiotics to treat vibriosis or other bacteria in aquaculture causes considerable harm and contributes to the development of antibiotic-resistant bacteria and to sustainable environmental water pollution. In addition, the microbiota diversity of water microorganisms could be damaged unrecoverably. 

Apart from application in animal health, phage application in bio-control against plant pathogenic bacteria is also generally widely discussed but so far there is no broad-scale or field application despite phytopathogens posing significant large-area problems and economic losses. This is also the case for potato diseases caused by *Pectobacterium atrosepticum* belonging to the *Enterobacteriaceae*. A specific phage has been found and in-depth genomically characterized by Buttimer et al., forming a new phage species of the genus *Certrevirus* [11]. The article illustrates the exciting unexpected complexity of phage genomic organization by means of comparative genomics and proteomics so that it allows insight into phage genome structure–function relationships.

Those analyses, together with studies on different aspects of phage–host interactions, such as defense mechanisms, will give valuable insights into phage biology and will contribute to the improvement of phage application. *Pseudomonas aeruginosa* is constantly a topic in infectiological research as this pathogen has evolved versatile defense mechanisms to resist antibacterials while it is a very widespread, very adaptable organism known to be obstinate at many sites of infection in the body persisting in biofilms. Pourcel et al. focus on the mechanisms of phage resistance of *P. aeruginosa*, which makes up to 10% of clinical strains by comparing phage-sensitive and phage-resistant strains [12]. The authors found a remarkable genomic flexibility in the latter with a large accessory genome consisting of different genomic elements. Furthermore, the major phage receptors in the lipopolysaccharide structure were affected, impeding phage adsorption and, interestingly, CRISPR-Cas systems could not be detected, confirming that they do not play a role in resistance to lytic phages whereas prophages conferring phage immunity and insertion elements were frequent. A very interesting finding was that multiphage-resistant *P. aeruginosa* isolates obviously have selective advantages, possibly to eliminate closely related strains. The authors postulate that the genomic complexity of *P. aeruginosa* with phage-sensitive and phage-resistant strains perhaps regulates cell density in vivo.

Besides analysis of resistance development for future application, it is of utmost significance to select the most suitable phage entities and to design optimal applicative conditions including phage dosage finding, therefore pre-clinical tests are highly relevant. Postulated there must be a phage–host-specific temporal evolution and population dynamics and hence, a kinetic fingerprint during phage infection of a given bacterial population. Aside from phage–host interaction and binding affinity characteristics, the multiplicity of infection (MOI) should have crucial influence. To understand the dynamics of bacterial and phage populations, an approach was developed by Loessner et al. [13] that allows one to understand the dynamics in terms of certain thresholds in bacterial and phage population sizes using the a. m. phage–host system: The proliferation threshold, SP, defining a minimal bacterial concentration required to support phage proliferation and the inundation threshold, VI, defining the phage concentration required to deplete the bacterial population. Despite the fact that predictions on the later efficacy and success of a phage therapy cannot be made built on in vitro experiments and not even on pre-clinical animal experiments, both are essential.

In light of the current pandemic, phages are in the focus again, apart from anti-bacterial purposes. Mass-produced vaccines producible within a few weeks are needed in a virus pandemic and the vaccine response to emerging RNA viruses like SARS-CoV-2 might be improved by using phages. Serwer [14] suggests applying coronavirus-like phages as pathogen homologs for developing model platforms; phages could be evolved that bind to specific antibodies against membrane-covered RNA viruses. A kind of vaccination could be induced by generating a localized non-hazardous bacterial infection with the phage host followed by applying the evolved phage which propagates within its bacterial host. This would be a live vaccine with the effect of a post-vaccination (phage) propagation. Most anti-viral vaccines are attenuated, live virus or inactivated virus vaccines. The author hypothesizes that the platform he discusses might ideally deliver more than enough to vaccinate everyone in the world with sufficient phage dose, if the evolved phages can be produced to propagate post-vaccination and suggests that virus phage homologs could be evolved if the antibodies are available. An antibody-selected SARS-CoV-1 phage homolog might serve to develop the SARS-CoV-2 homolog, concluding that inter-pandemic activity would be needed to improve vaccines for current pathogens.

Apart from the applicative purposes, but directly linked to phage diversity, Wittmann and other members of the International Committee on Taxonomy of Viruses (ICTV) report the current taxonomic reassessment of a large and diverse group of N4-like viruses and present new insights into the diversity and taxonomy of phages [15]. Due to new and cheaper sequencing methods, the number of available genome sequences in public databases is enormously increasing. However, most of them stay taxonomically unclassified despite the high efforts of the ICTV. This brief report describes the current methodology for classifying phages and the changes from long-lasting morphological approaches to using genomics and proteomics. Based on those approaches and findings, the authors propose to create a new family “*Schitoviridae*” including eight new subfamilies and numerous new genera.
